# RNA-seq Based Transcriptome Analysis Reveals The Cross-Talk of Macrophage and Adipocyte of Chicken Subcutaneous Adipose Tissue during The Embryonic and Post-Hatch Period

**DOI:** 10.3389/fimmu.2022.889439

**Published:** 2022-07-15

**Authors:** Haidong Zhao, Mingli Wu, Xiaoqin Tang, Qi Li, Xiaohua Yi, Wanxia Zhao, Xiuzhu Sun

**Affiliations:** ^1^ College of Animal Science and Technology, Northwest A&F University, Yangling, China; ^2^ College of Grassland Agriculture, Northwest A&F University, Yangling, China

**Keywords:** chick, subcutaneous adipose tissue, abdominal adipose tissue, FFAs, macrophage, adipocyte

## Abstract

With high fecundity and short production cycle, poultry is one of the important sources of meat. During the embryonic and post-hatch period, the higher death rate caused huge economic losses in poultry production. Our previous study showed that chick subcutaneous adipose tissue is an important energy supply tissue besides yolk. Therefore, the metabolic mechanism of subcutaneous adipose tissue in chicks could provide a new perspective of brooding. The objectives of the current study were to evaluate the differences between chick subcutaneous adipose tissue and abdominal adipose tissue before and after hatching and reveal the cross-talk of different cells within the chick subcutaneous adipose tissue. The results of RNA-seq and weighted gene co-expression network analysis (WGCNA) of chick subcutaneous and abdominal adipose tissues showed that the function of chick subcutaneous tissue was related to immunoreaction, and macrophage could be the major immune infiltration cell type in chicken subcutaneous adipose tissue, which were also verified by qPCR, HE stain, and IHC. The results of free fatty acids (FFAs)-induced the cross-talk between macrophages and adipocytes showed that FFAs-*Ccl2* (chicken *CCL26*) axis could have an important role in lipid transportation in adipose tissue. The results of Oil Red O and Nile red stain demonstrated that macrophages have the ability to absorb FFAs quickly. Interestingly, according to the genomic organization of *CCL* family with representative vertebrate species, we found that chicken *CCL26* could be the major chemokine in chicken adipocyte as the status of *CCL2* in mammal adipocyte. In conclusion, we demonstrate that FFA-induced *Ccl2* (chicken *CCL26*) secretion is crucial in determining fat depot-selective adipose tissue macrophage (ATM) infiltration, which could be an important medium of lipid transportation in chicken subcutaneous adipose tissue. These findings may have multiple important implications for understanding macrophage biology with chick subcutaneous adipose tissue and provide theoretical basis for lipid metabolism in poultry brooding.

## Introduction

Poultry is one of the important sources of meat, with high fecundity, short production cycle, and other advantages. The first few days after hatching, high mortality has brought huge economic losses to the poultry industry. Therefore, it is of great significance to understand the metabolic mechanism of this special development stage. At hatch period, the main energy supply of chicks is lipid, which mainly exists in unabsorbed yolk and subcutaneous adipose tissue. The storage of subcutaneous adipose tissue in chicks is much higher than that of abdominal adipose tissue, suggesting that subcutaneous adipose tissue plays an important function after hatching. However, the metabolism dynamics of chick subcutaneous adipose tissue is still poorly understood at present. The results of our previous study found that the subcutaneous adipose tissue of chicks experienced great fluctuations from embryonic stage to post-hatch stage, but the metabolic mechanism of subcutaneous adipose tissue in chicks remains to be further explored.

The adipose tissue is a major site of energy storage and maintains systemic metabolic homeostasis through taking up and hydrolyzing lipid (1). Understanding the molecular mechanism of adipose tissue in physiological and pathological settings has important implications to cope with human health and animal husbandry. The excessive lipid deposition is not the specific cause of lipometabolic disturbance, and their regulator network is complex. First, lipid homeostasis depends on the involvement of adipose tissue and the digestive, nervous, endocrine, and immune systems (2, 3). Second, there are clear metabolic differences among adipose tissues distributed in different parts (4, 5). Moreover, the heterogeneity of adipose tissue and the cross-talk of different cell types in adipose tissue maintain lipid homeostasis as a most important mechanism (6–8).

The adipose tissue is divided into white adipose tissue (WAT) and brown adipose tissue (BAT) (9). BAT exerts a protective role of non-shivering thermogenesis by fatty acid oxidation and energy metabolism by glucose utilization (10). The existence of BAT in birds has not been determined, but the deletion of the *uncoupling protein 1* (*UCP1*) gene is a major defect in the birds’ non-shivering thermogenesis (11). Whether there exists WAT browning in birds is also lacking scientific basis (12). The results of our previous study showed that peroxisome proliferator-activated receptor gamma (PPARγ) and carnitine palmitoyltransferase 1A (CPT1a) could be the key points of fatty acid oxidation and non-shivering thermogenesis in chick subcutaneous adipose tissue after hatching (13). At the same time, it has caught our attention why there are a lot of depositions of subcutaneous adipose tissue rather than abdominal adipose tissue during the chick embryo period. In recent years, studies have shown that there were differences between adipose tissue present in subcutaneous adipose tissue and visceral adipose tissue present in the abdominal cavity (14, 15). There are more glucocorticoid and steroid hormone receptors in the visceral adipose tissue than in the subcutaneous adipose tissue, while the subcutaneous adipose tissue is more avid in absorption of circulating free fatty acids and triglycerides (16, 17). It is possible that the function of vast amounts of subcutaneous adipose tissue in chick before hatching could be related to the absorption of remnant yolk. In this respect, understanding the lipid dynamics of subcutaneous adipose tissue of chicks has great significance for yolk absorption and lipid metabolism before and after hatching.

As a heterogeneous tissue, a variety of cell types are present within the adipose tissue, including macrophages, mast cells, neutrophils, eosinophils, group 2 innate lymphoid cells, T cells, B cells, vascular stromal cells, and neurocytes. The cross-talk of different cells within the adipose tissue is the biological foundation of lipid homeostasis (18–20). Recent studies showed that the cross-talk of macrophages and adipocytes generally participates in lipoinflammation (21, 22). When confronted with the metabolic stress after hatching, adipocytes recruit macrophages in response to inflammatory signals for keeping lipid homeostasis. The medium of cross-talk between adipocytes recruiting macrophages is complicated and changeable, including adipocytokines, fatty acid, chemokines, inflammation cytokines, and extracellular vesicles (23–26). However, there is no report related to the cross-talk of adipocytes and macrophages in chick subcutaneous adipose tissue at present.

Therefore, the objectives of current study were to evaluate the differences between chick subcutaneous adipose tissue and abdominal adipose tissue before and after hatching through RNA-seq and weighted gene co-expression network analysis (WGCNA), and to explore the cross-talk between adipocytes and macrophages in lipoinflammation and lipid homeostasis, aiming to provide theoretical basis on physiological characteristics about lipid metabolism and brood in poultry production.

## Materials and methods

All experimental procedures were performed in accordance with the Regulations for the Administration of Affairs Concerning Experimental Animals approved by the State Council of the People’s Republic of China. The study was approved by the Institutional Animal Care and Use Committee of Northwest A&F University (Permit Number NWAFAC1019).

### Animals

Lohmann pink chicken embryos and chicks used in the current study were bought from the Yangling Julong Poultry Industry Co. Ltd. (Yangling, China). Incubation conditions were as follows: E (embryonic) 1–E19, 37.8°C, E19–E21, 37°C–37.5°C (Qingdao Xinyi Electronic Equipment Co., Ltd., Qingdao, China). After hatching, chicks were allowed *ad libitum* access to water and feed. Chicks were humanely euthanized by cervical dislocation, and subcutaneous adipose tissues were collected. Considering that the chicken embryos have individual differences, 12 subcutaneous adipose tissue samples were collected in four development stage (E14, E20, D1, and D9). Samples for RNA and protein detection were stored at −80°CC, and the other samples for paraffin section were dipped in 4% formaldehyde until analysis. Three adult Lohmann pink chickens used for macrophage isolation were also purchased from the Yangling Julong Poultry Industry Co. Ltd. (Yangling, China).

### RNA Isolation and Library Preparation

Total RNA for RNA-seq was extracted using RNAiso plus kit (Takara, Tokyo, Japan) from 12 samples in four stages (E14, E20, D1, and D9). Total RNA concentrations were evaluated using Qubit 2.0 kit (Invitrogen, CA, USA), and total RNA quality were evaluated using agarose electrophoresis (Sangong, Shanghai, China). Twelve RNA samples were mixed into three RNA pools of equal mass in every development stages (E14, E20, D1, and D9). Twelve complementary DNA (cDNA) libraries were prepared using Hieff NGS™ MaxUp Dual-mode messenger RNA (mRNA) Library Prep Kit for Illumina^®^ (YEASEN Biotech Co., Ltd, Shanghai, China). The quality of cDNA libraries was measured by Qubit 2.0 fluorometer DNA assay kit (Invitrogen, CA, USA) and submitted for sequencing (Illumina Xten, San Diego, USA).

### Primer Design and qPCR

qPCR was performed to verify the RNA-seq expression pattern of different development stages. Total RNA for quantitative real-time PCR (qPCR) was extracted using RNAiso plus kit (Takara, Tokyo, Japan) from 12 samples in four stages (E14, E20, D1, and D9). Total RNA concentration and quality were evaluated using Nanodrop 1000 (Thermo, MA, USA). Reverse transcription of RNA to cDNA (Takara, Tokyo, Japan) was performed before qPCR, carried out in the Y480 Real-Time PCR detection system (Roche, Basel, Switzerland) utilizing SYBR green detection (Takara, Tokyo, Japan). Primers designing used NCBI primer, and their Tm was close to 60°C (**Supplementary Table S1**). The amplification protocol was as follows: 95°C for 30 s, followed by 50 cycles of 95°C 10 s and 60°C for 30 s. Melt curve analysis was performed between 55°C and 95°C, with a 0.5°C increment every 5 s. Samples were run in triplicate. All mRNA expression levels were normalized to the arithmetic mean of ACTB (27); the mRNA relative expression was quantified using the 2^−ΔΔCt^ method (28). Twelve differentially expressed genes (DEGs) [fold change (FC) > 2 and false discovery rate (FDR) < 0.05] were randomly chosen for verification by qPCR. Hub genes belonging to DEGs were also chosen for verification by qPCR.

### HE Staining, Toluidine Blue Staining, and IHC

Chick subcutaneous adipose tissues were fixed in 4% paraformaldehyde solution in PBS for 2 days and processed through a series of procedures including dehydration, paraffin embedding, sectioning, and staining. All these procedures were performed by Wuhan Servicebio Technology Co., Ltd. (Wuhan, China), including hematoxylin and eosin (HE), toluidine blue, and immunohistochemistry (IHC). Primary antibody information was LGALS3 (Galectin 3) (Servicebio, Wuhan, China).

### Cell Culturing

#### Chicken Adipocyte Culturing

Chick embryos were sacrificed by cervical dislocation, and the subcutaneous adipose tissue was placed in a 10-ml tube with 1 ml phosphate-buffered saline (PBS) at room temperature, cut into tiny bits with scissors. Adipocyte was obtained by collagenase II digestion (Sangong, Shanghai, China). The condition was as follows: 0.1 mg/ml collagenase II in 1× Hanks’ balanced salt solution, incubated at 37°C for 1 h and shook every 5 min. Adipocyte was collected from cell suspension through a 200-mesh cell strainer and maintained in Dulbecco’s modified Eagle’s medium (DMEM) high glucose containing 10% fetal bovine serum (FBS) (Solarbio Science & Technology Co., Ltd., Beijing, China), 5% chicken serum (Solarbio Science & Technology Co., Ltd, beijing, China), 50 U/ml penicillin, and 50 μg/ml streptomycin (Sangong, Shanghai, China).

#### Chicken Macrophage Culturing

Chicken-monocyte-derived macrophages were collected in chicken blood by density gradient separation (Solarbio Science & Technology Co., Ltd., Beijing, China) and maintained in Roswell Park Memorial Institute (RPMI) 1640 containing 10% FBS, 5% chicken serum, 50 U/ml penicillin, and 50 μg/ml streptomycin.

RAW264.7 macrophage was maintained in RPMI 1640 containing 10% FBS, 50 U/ml penicillin, and 50 μg/ml streptomycin.

3T3-L1 adipocyte was maintained in DMEM high glucose containing 10% FBS, 50 U/ml penicillin, and 50 μg/ml streptomycin.

Three free fatty acids (FFAs) [palmitic acid (PA), oleic acid (OA), and linoleic acid (LA)] (Sangong, Shanghai, China) principally in adipose tissue and yolk were used in this study for evaluating the effect of the adipose tissue macrophage (ATM) infiltration.

### Western Blot

Cells were collected for protein experiments after FFA treatment. Total protein of adipocyte was lysed with radioimmunoprecipitation assay (RIPA) lysis buffer (1 mM MgCl_2_, 10 mM Tris–HCl pH 7.4, 1% Triton X-100, 0.1% sodium dodecyl sulfate (SDS), and 1% Nonidet P40 cocktail). The proteins were collected and quantified by using the BCA™ Protein Assay Kit (Sangon, Shanghai, China). The proteins were separated by 5%–12% SDS–polyacrylamide gel electrophoresis (SDS-PAGE) and transfected to polyvinylidene fluoride (PVDF) membranes and blocked in 5% non-fat milk for 1 h at room temperature. The membranes were incubated overnight with the following primary antibodies: CCL2 (C–C motif chemokine ligand 2) (Sangon, Shanghai, China), CCL5 (Beyotime, Shanghai, China), CCR2 (C–C motif chemokine receptor 2) (Sangon, Shanghai, China), and CCR5 (Sangon, Shanghai, China). Then, blots were washed three times with PBS and were incubated with horseradish peroxidase (HRP)-conjugated goat anti-rabbit IgG (Sangon, Shanghai, China) for 1 h at room temperature. The blots were examined by using ECL reagents (Sangon, Shanghai, China) according to the manufacturer’s instructions. The intensity of the bands was quantified by using Image Lab™ Software (Bio-Rad).

### Cell Migration and Recruitment Assays

FFA-induced cell migration between adipocyte and macrophage was detected by wound healing insert (ibidi, Gräfelfing, Germany). In brief, cell density of adipocyte and macrophage was adjusted to 1×10^5^ 3T3-L1. Then, a total of 100 μl 3T3-L1 adipocyte and RAW264.7 macrophage suspension was respectively added into the wound healing insert. After removing the attachment, the wound healing insert was removed and counted microscopically (Shunyu, Ningbo, China). After incubation for 24 h in three FFAs (100 μM) condition medium, the 0 μM FFA complete medium was used as negative control (NC). Cells were fixed in 4% paraformaldehyde solution and counted microscopically again. FFA-induced cell recruitment between 3T3-L1 adipocyte and RAW264.7 macrophage was detected by transwell assay with a pore size of 12 μm (NEST Biotechnology, Wuxi, China). In brief, 3T3-L1 adipocyte and RAW264.7 macrophage were washed twice with PBS and resuspended in the medium and adjusted to suit cell density. Then, RAW264.7 macrophage suspension was added to the upper compartment of a six-well transwell culture chamber. 3T3-L1 adipocyte suspension in three FFAs (100 μM) condition medium was added to the undercompartment of the six-well transwell culture chamber. After incubation for 24 h, cells were fixed in 4% paraformaldehyde solution, stained with 1× Wright-Giemsa staining solution (Sangon, Shanghai, China), and counted microscopically again. The processing of cell migration and recruitment between chicken adipocyte and macrophage was same as abovementioned.

### Oil Red O and Nile Red Staining

RAW264.7 and chicken macrophages were cultured in three FFAs (100 μM) condition medium for 24 h. Oil red and Nile red staining were used to detect the macrophage foaming processing. For Oil red O staining, samples were fixed for 30 min in 4% paraformaldehyde. After washing, fixed cells were incubated in Oil red O (Sangong, Shanghai, China) staining solution (0.5% in isopropanol) for 20 min. For Nile red staining, samples were fixed in 4% paraformaldehyde for 30 min. After washing, fixed cells were incubated for 5 min in Nile red (Sangong, Shanghai, China) staining solution and counter-stained in Hoechst 33342 staining solution (Sangong, Shanghai, China).

### RNA-seq Analysis and Statistics

Fast QC (http://www.bioinformatics.babraham.ac.uk/projects/fastqc/) was used for quality control (29). Trimmomatic (http://www.usadellab.org/cms/?page=trimmomatic) was used to remove reads containing adapter, reads containing poly-N, and low-quality reads (30). Q20 was the filter standard for the following analysis. Available data were mapped to the reference genome sequence from NCBI database (GCF_016699485.2) using HISAT2 tool (https://daehwankimlab.github.io/hisat2/) (31). DEGs were determined by DESeq2 (32) and satisfy the fold change >2 and FDR <0.05. Gene Ontology (GO) enrichment analysis and Kyoto Encyclopedia of Genes and Genomes (KEGG; https://www.kegg.jp/) pathway enrichment analysis were performed using DAVID (http://david.abcc.ncifcrf.gov/) and satisfy the condition that FDR <0.05. Paired t-test was performed for qPCR verification of DEGs and hub genes by SPSS 18.0 (IBM, New York, USA). For weighted gene co-expression network analysis (WGCNA) (33), more than 90% of the genes with counts <10 were removed, and the median absolute deviation (MAD) of top 5,000 genes was extracted to construct a coexpression network. WGCNA parameters were as follows: power=8, minModuleSize=30, networkType=“signed,” corType=“pearson,” TOMType=“signed,” and mergeCutHeight=0.25. The functional enrichment analysis of genes in each module was performed by DAVID dataset (http://david.abcc.ncifcrf.gov/). The protein–protein interaction (PPI) network of the interested module was constructed using the STRING online database (https://string-db.org/) (34). The degree algorithm of Cytohubba plugin based on Cytoscape was used to identify the high-degree genes, which played a critical role in the PPI (35).

### Statistics

Statistical analysis was performed using SPSS software version 21.0 (SPSS Inc., Chicago, IL) or Microsoft Excel (Microsoft). Data analysis involved unpaired two-tailed Student’s t-test for two groups and one-way ANOVA for more than two groups. Data shown are average ± standard error of the mean (SEM). The *p*-value of <0.05 was considered to be statistically significant.

## Results

### RNA-seq Library Characteristics of Subcutaneous Adipose Tissue During the Embryonic and Post-Hatch Period

**Figure 1:**
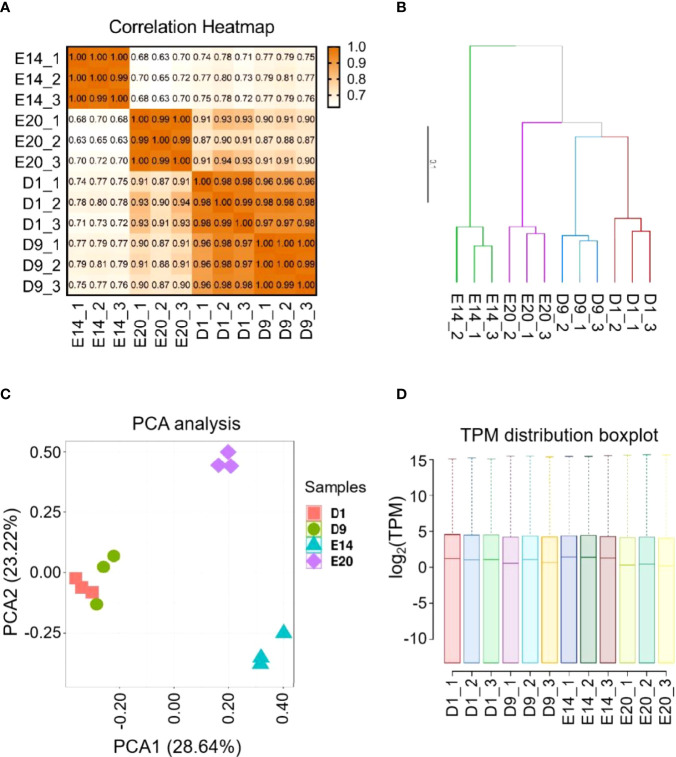
RNA-Seq library characteristics of chick subcutaneous adipose tissue in four development stages. **(A)** Correction analysis of 12 RNA-Seq library of chick subcutaneous adipose tissue in four development stages; **(B)** cluster analysis of 12 RNA-Seq library of chick subcutaneous adipose tissue in four development stages; **(C)** PCA of 12 RNA-Seq library of chick subcutaneous adipose tissue in four development stages; **(D)** TPM distribution box plot of 12 RNA-Seq library of chick subcutaneous adipose tissue in four development stages.

Twelve libraries represented four development stages, including E14, E20, D1, and D9. The range of total reads for the 12 samples was 40,146,820–56,033,980. For each sample, the number of total mapped reads were more than 94%, the number of uniquely mapped reads were more than 92%, the Q20 bases ratio were more than 96%, and Q30 bases ratio were more than 87% (**Supplementary Table S2**). The results of correlation and clustering analyses showed that 12 samples were divided into four groups, which were the same as their development stages (**Figures 1A, B** **)**. The result of the principal component analysis (PCA) was similar to that of the correlation analysis, but D1 and D9 groups were indistinguishable (**Figures 1C**). The distribution of TPM was similar across all samples (**Figure 1D**).

### Expression Dynamics of mRNAs in the Subcutaneous and Abdominal Adipose Tissues During the Embryonic and Post-Hatch Period

**Figure 2:**
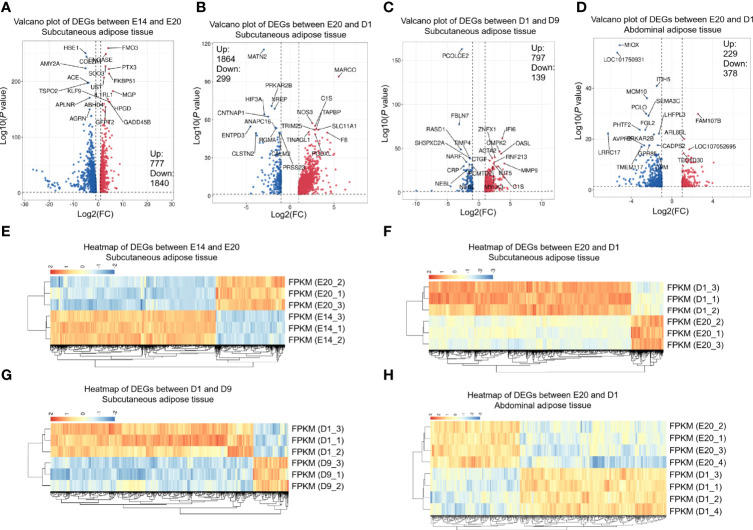
DEGs between different development stages of chick subcutaneous adipose tissue and abdominal adipose tissue. **(A)** Volcano of DEGs between E14 and E20 in chick subcutaneous adipose tissue; **(B)** volcano of DEGs between E20 and D1 in chick subcutaneous adipose tissue; **(C)** volcano of DEGs between D1 and D9 in chick subcutaneous adipose tissue; **(D)** volcano of DEGs between D1 and D9 in chick abdominal adipose tissue; **(E)** heatmap of DEGs between E14 and E20 in chick subcutaneous adipose tissue; **(F)** heatmap of DEGs between E20 and D1 in chick subcutaneous adipose tissue; **(G)** heatmap of DEGs between D1 and D9 in chick subcutaneous adipose tissue; **(H)** heatmap of DEGs between E14 and E20 in chick abdominal adipose tissue.

**Figure 3:**
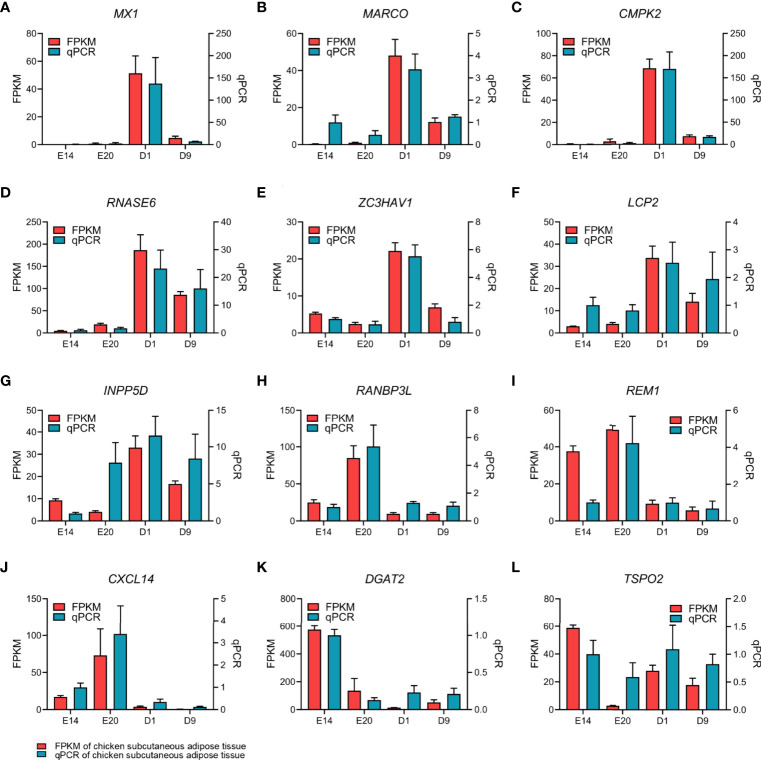
qPCR verification of 12 EDGs between E20 and D1 in chick subcutaneous adipose tissue in four development stages.

Pairwise comparisons between the subcutaneous adipose tissue libraries in four development stages from embryo or chick were used to examine mRNA expression. Between E14 and E20, 777 genes were upregulated, and 1,840 genes were downregulated (**Figures 2A, E** **)**. Between E20 and D1, 1,864 genes were upregulated, and 299 genes were downregulated (**Figures 2B, F** **)**. Between D1 and D9, 797 genes were upregulated, and 139 genes were downregulated (**Figures 2C, G** **)**. The cutoff criterion of DEGs were FDR <0.05 and log2|FC|>1. All the DEGs between different development stages are shown in **Supplementary Table S3**. Twelve DEGs were selected randomly for verification by qPCR. Eleven DEGs showed similar expression dynamics as RNA-seq data (*MX1*, *MARCO*, *CMPK2*, *RNASE6*, *ZC3HAV1*, *LCP2*, *INPP5D*, *RANBP3L*, *REM1*, *CXCL14*, *DGAT2*, and *TSPO2*) (**Figures 3A–L**). Undeniably, the mRNA expression dynamics of some genes were not completely the same as the RNA-seq in each developmental stage. In addition, DEGs of the abdominal adipose tissue between different development stages were referred to a previous study (36). Moreover, DEGs of the abdominal adipose tissue between E20 and D1 were analyzed by DESeq2 R-package, which had 229 upregulated genes and 378 downregulated genes (**Figures 2D, H** **)**.

### Enrichment Analysis of the Subcutaneous and Abdominal Adipose Tissues During the Embryonic and Post-Hatch Period

**Figure 4:**
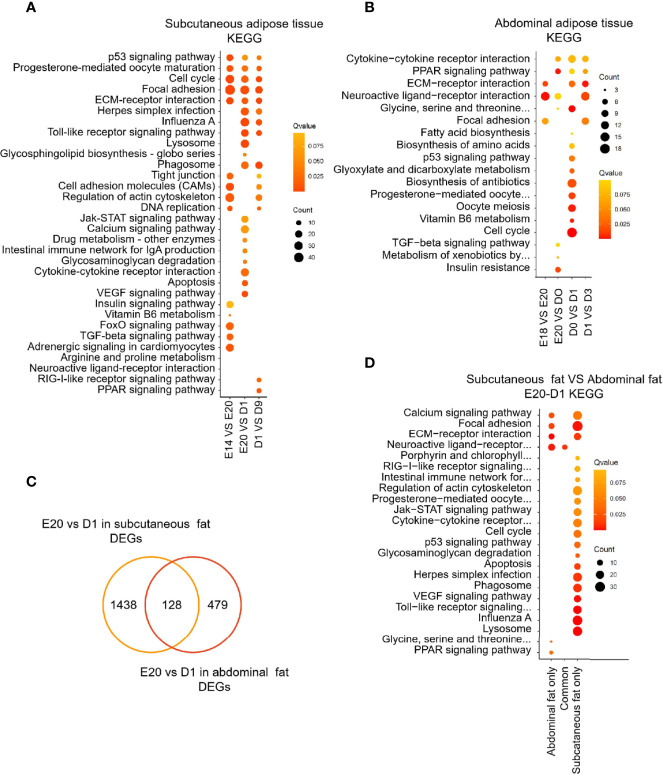
KEGG enrichment of DEGs between different development stages in chick subcutaneous and abdominal adipose tissues. **(A)** KEGG enrichment of DEGs between different development stages in chick subcutaneous adipose tissue in four development stages; **(B)** KEGG enrichment of DEGs between different development stages in chick abdominal adipose tissue in five development stages; **(C)** Venn analysis of DEGs between subcutaneous adipose tissue and abdominal adipose tissue (E20 vs. D1); **(D)** KEGG enrichment of Venn analysis of DEGs between subcutaneous adipose tissue and abdominal adipose tissue (E20 vs. D1).

**Figure 5:**
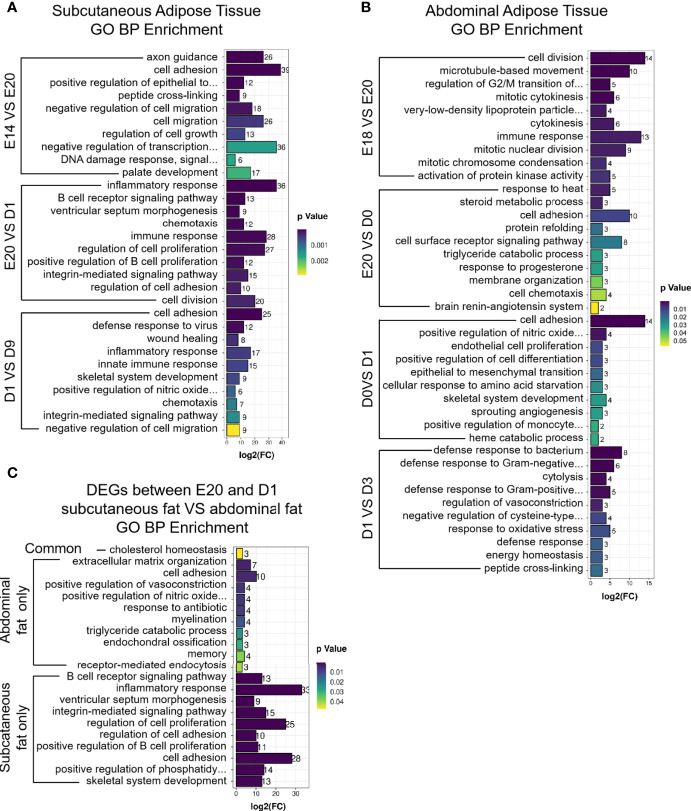
GO enrichment of DEGs between different development stages in chick subcutaneous and abdominal adipose tissues. **(A)** GO enrichment of DEGs between different development stages in chick subcutaneous adipose tissue; **(B)** GO enrichment of DEGs between different development stages in chick abdominal adipose tissue; **(C)** GO enrichment of Venn analysis of DEGs between subcutaneous adipose tissue and abdominal adipose tissue (E20 vs. D1).

GO and KEGG analyses were performed to determine the biological function of DEGs between different development stages in chick subcutaneous and abdominal adipose tissues. KEGG analysis of DEGs was divided into three parts, including subcutaneous adipose tissue, abdominal adipose tissue, and comparison of subcutaneous and abdominal adipose tissues. For chick subcutaneous adipose tissue, the results of KEGG enrichment between E14 and E20 showed that DEGs were involved in cell proliferation and tissue development, the results of KEGG enrichment between E20 and D1 showed that the function of DEGs were associated with immune response and inflammation, and the results of KEGG enrichment between D1 and D9 showed that DEGs were related to adipocyte differentiation (**Figure 4A**; **Supplementary Table S4**). For chick abdominal adipose tissue, the results of KEGG enrichment between E18 and E20 showed that DEGs were involved in neuroactive ligand receptor and focal adhesion. The results of KEGG enrichment between E20 and D0 showed that DEGs were involved in insulin resistance and PPAR signaling pathway. The results of KEGG enrichment between D0 and D1 showed that DEGs were involved in cell proliferation and apoptosis. The results of KEGG enrichment between D1 and D3 showed that DEGs were involved in PPAR signaling pathway (**Figure 4B**; **Supplementary Table S5**). Comparing the DEGs (E20 vs. D1) between subcutaneous adipose tissue and abdominal adipose tissue (**Supplementary Table S6**), the functions of DEGs in subcutaneous adipose tissue were centered on immunity and inflammation, and the functions of DEGs in abdominal adipose tissue were related to PPAR signaling pathway (**Figures 4C, D**; **Supplementary Table S7**). The results of GO enrichment were similar to KEGG enrichment; the function of DEGs (E20 vs D1) in subcutaneous adipose tissue was related to immune and inflammation, and the function of DEGs (E20 vs D1) in abdominal adipose tissue was involved in adipocyte differentiation (**Figure 5**).

### WGCNA and Hub Genes Identification of the Subcutaneous and Abdominal Adipose Tissues During the Embryonic and Post-Hatch Period

**Figure 6:**
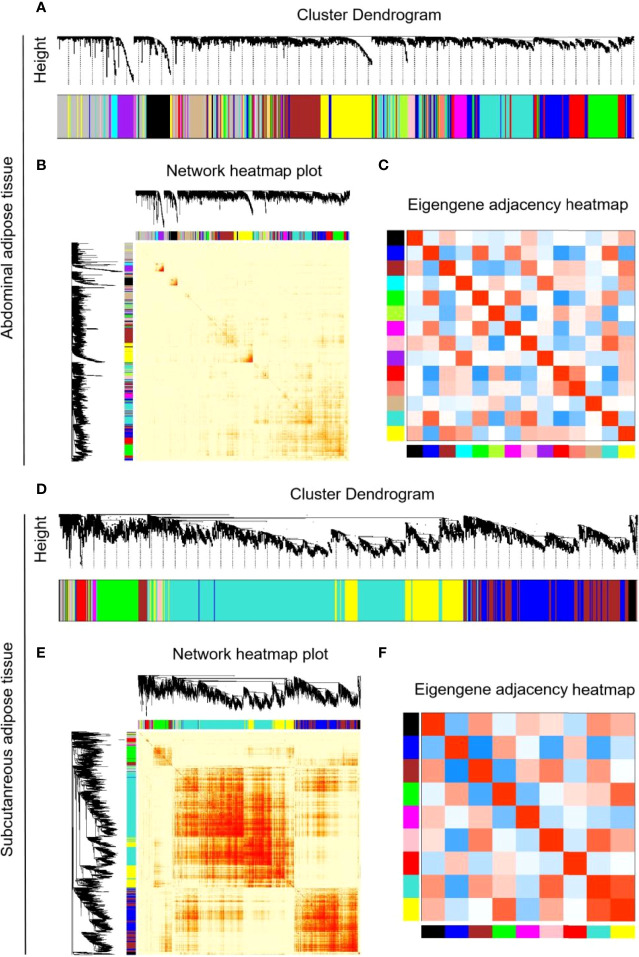
WGCNA parameters of RNA-Seq library of chick subcutaneous and abdominal adipose tissues from embryonic to post-hatch development. **(A)** Cluster dendrogram of RNA-Seq library of chick abdominal adipose tissue in five development stages; **(B)** network heatmap of RNA-seq library of chick abdominal adipose tissue in five development stages; **(C)** eigengene adjacency heatmap of RNA-seq library of chick abdominal adipose tissue in five development stages; **(D)** cluster dendrogram of RNA-seq library of chick subcutaneous adipose tissue in four development stages; **(E)** network heatmap of RNA-seq library of chick subcutaneous adipose tissue in four development stages; **(F)** eigengene adjacency heatmap of RNA-Seq library of chick subcutaneous adipose tissue in four development stages.

**Figure 7:**
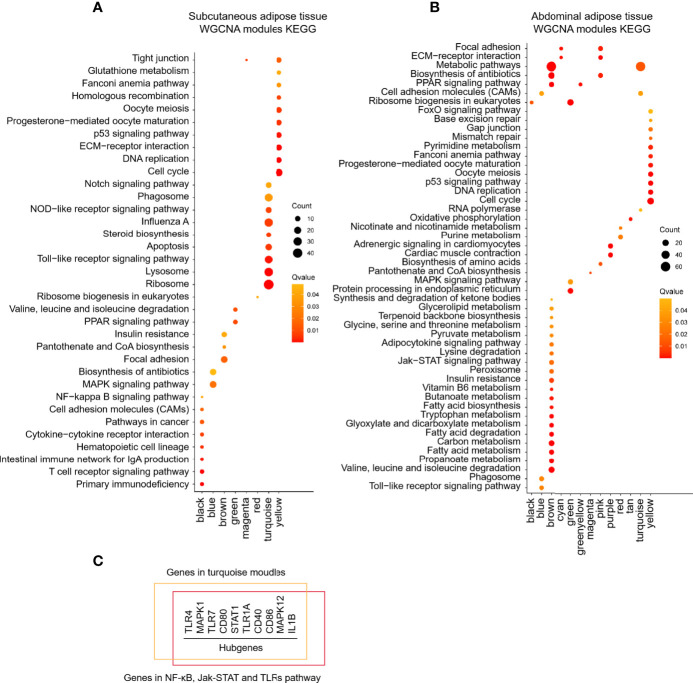
KEGG enrichment of different modules of chick subcutaneous and abdominal adipose tissues from embryonic to post-hatch development by WGCNA. **(A)** KEGG enrichment of different modules of chick subcutaneous adipose tissue in four development stages by WGCNA; **(B)** KEGG enrichment of different modules of chick abdominal adipose tissue in five development stages by WGCNA; **(C)** hub genes identification of turquoise module in chick subcutaneous adipose tissue in four development stages.

**Figure 8:**
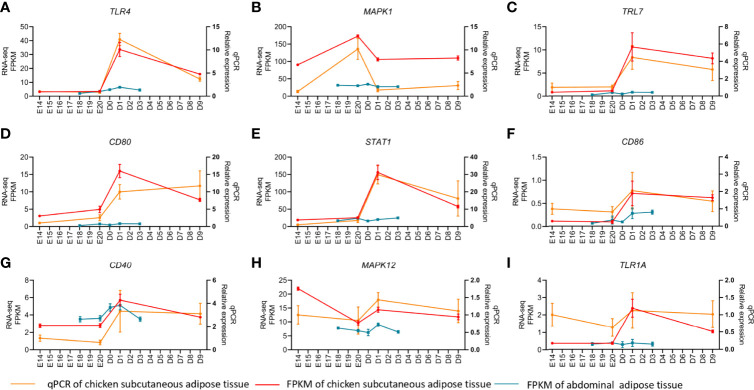
qPCR verification of hub genes in chick subcutaneous and abdominal adipose tissues during the peri-hatching period in chickens.

MADs of the top 5,000 genes were used to construct a coexpression network from different development stages of subcutaneous and abdominal adipose tissues, respectively. The MADs of the top 5,000 genes of the abdominal adipose tissue were clustered into 14 modules, including black (n=210), blue (n=575), brown (n=491), cyan (n=52), green (n=459), greenyellow (n=147), magenta (n=164), pink (n=187), purple (n=161), red (n=404), salmon (n=79), tan (n=147), turquoise (n=695), and yellow (n=486). The MADs of the top 5,000 genes of the subcutaneous adipose tissue were clustered into nine modules, black (n=68), blue (n=823), brown (n=712), green (n=365), magenta (n=35), pink (n=45), red (n=96), turquoise (n=2,056), and yellow (n=700). All the genes of different modules from subcutaneous and abdominal adipose tissues are shown in **Supplementary Table S8**. The results of cluster dendrogram, network heatmap, and eigengene adjacency indicated a significant difference among modules from subcutaneous and abdominal adipose tissues, respectively (**Figure 6**). According to the results of KEGG enrichment analysis from each module, the functions of subcutaneous adipose tissue in the turquoise module were involved in lysosome, toll-like receptor signaling pathway, and apoptosis (**Figure 7A**), and the function of the abdominal adipose tissue in the brown module was related to fatty acid metabolism (**Figure 7B**). The results of GO and KEGG enrichment analyses of all modules are shown in **Supplementary Tables S9**, **S10**. The hub genes of subcutaneous adipose tissue turquoise module were obtained from the intersection of genes in the turquoise module and genes inflammation pathway (NF-κB, Jak-STAT, and TLR pathways) (**Figure 7C**). All the hub genes in the subcutaneous adipose tissue were verified by qPCR, and their mRNA expression dynamics was similar to RNA-seq in the subcutaneous adipose tissue and different from RNA-seq data in the abdominal adipose tissue from embryonic to post-hatch period (**Figures 8A–I**).

### Immune Cells Infiltration and Related Genes Expression of Subcutaneous Adipose Tissue During the Embryonic and Post-Hatch Period

**Figure 9:**
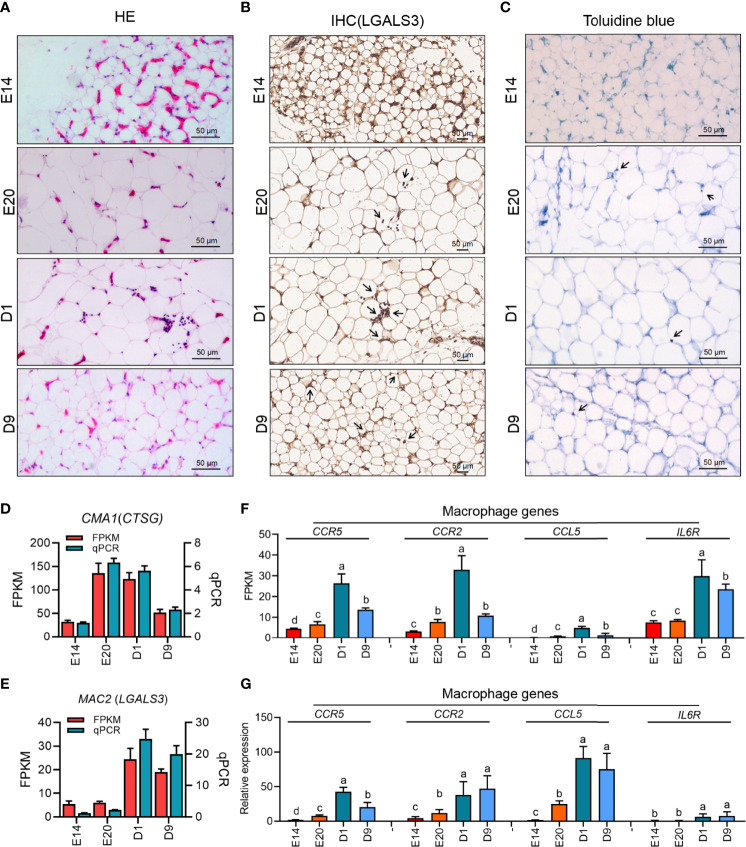
Immune infiltration of four development stages of subcutaneous adipose tissue in chicken. **(A–C)** HE staining, IHC staining (LGALS3), and toluidine blue staining of chick subcutaneous adipose tissue in four development stages; **(D, E)** the expression of mast cell (*CMA1*) and macrophage (*MAC2*) marker genes of chick subcutaneous adipose tissue in four development stages; **(F)** FPKM of macrophage-related genes of chick subcutaneous adipose tissue in four development stages; **(G)** qPCR of macrophage-related genes of chick subcutaneous adipose tissue in four development stages.

The results of HE staining of chicken subcutaneous adipose in E14, E20, D1, and D9 showed that the level of immune cells infiltration in D1 was higher than that in E20 (**Figure 9A, E** **)**. As the marker gene of chicken macrophage (lacking of F4/80), LGALS3 IHC staining and mRNA expression demonstrated that macrophage was the major type in chicken subcutaneous adipose tissue after hatching (**Figures 9B, D** **)**. To exclude mast- and T-cell infiltration effects, qPCR and toluidine blue staining were used to identify the level of mast and T cells. The results of toluidine blue and mast cells marker gene (*CTSG*) showed that there was lower mast cell infiltration in chicken subcutaneous adipose tissue during the embryonic and post-hatch period (**Figure 9C**). Besides, there was no signal of T-cell marker genes (*CD4* and *CD8A*) for qPCR examination in chicken subcutaneous adipose tissue samples. According to the evidence of immune cells infiltration of chicken subcutaneous adipose tissue, the cross-talk of macrophage and adipocyte could be crucial in determining lipid metabolism during the embryonic and post-hatch period. In addition, part of the important genes in adipocyte and macrophage was analyzed and showed once again the importance of cross-talk between macrophage and adipocyte (**Figures 9F, G** **)**.

### Chematic Structure of the Genomic Organization and Expression Pattern of CCL Family and Its Relatedness with Representative Vertebrate Species

**Figure 10:**
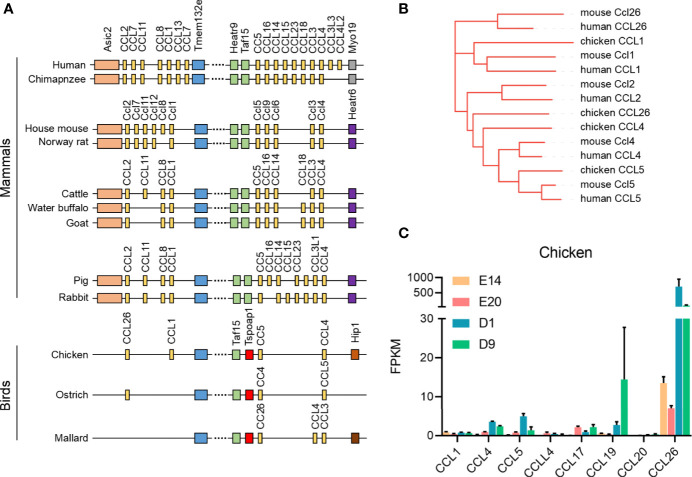
Schematic structure of the genomic organization and expression of CCL family with representative vertebrate species. **(A)** Schematic structure of the genomic organization of CCL family with representative vertebrate species; **(B)** the phylogenetic tree of CCL26, CCL1, CCL2, CCL4, and CCL5 in human, mouse, and chicken; **(C)** the expression pattern of CCL family of chicken subcutaneous adipose tissue in four development stages.

*CCL2* was the major chemokine of adipocyte in most mammals. Chickens do not have the *CCL2* gene, so we have investigated the structure of the genomic organization of *CCL2* and *CCL5* gene clusters in chicken and representative species. In mammals, the *CCL2* gene cluster was located in the upstream of *Tmem132e* gene, and the *CCL5* gene cluster was located in the downstream of *Tmem132e* gene. In birds, the locus of the mammal *CCL2* gene was the bird *CCL26* gene, suggesting that *CCL26* gene in birds could have similar function to *CCL2* gene in mammals (**Figure 10A**). Based on the phylogenetic tree of *CCL26*, *CCL1*, *CCL2*, *CCL4*, and *CCL5* in human, mouse, and chicken, the chicken *CCL26* has a closer kinship with *CCL2* rather than *CCL26* in human and mouse (**Figure 10D**). According to the mRNA expression pattern of *CCL* gene family in chicken adipose tissue, *CCL26* could be the major chemokine in chicken adipocyte as the status of *CCL2* in mammal adipocyte (**Figures 10B, C**).

### FFA-Induced Adipocyte and Macrophage Chemokines and Receptors Expression

**Figure 11:**
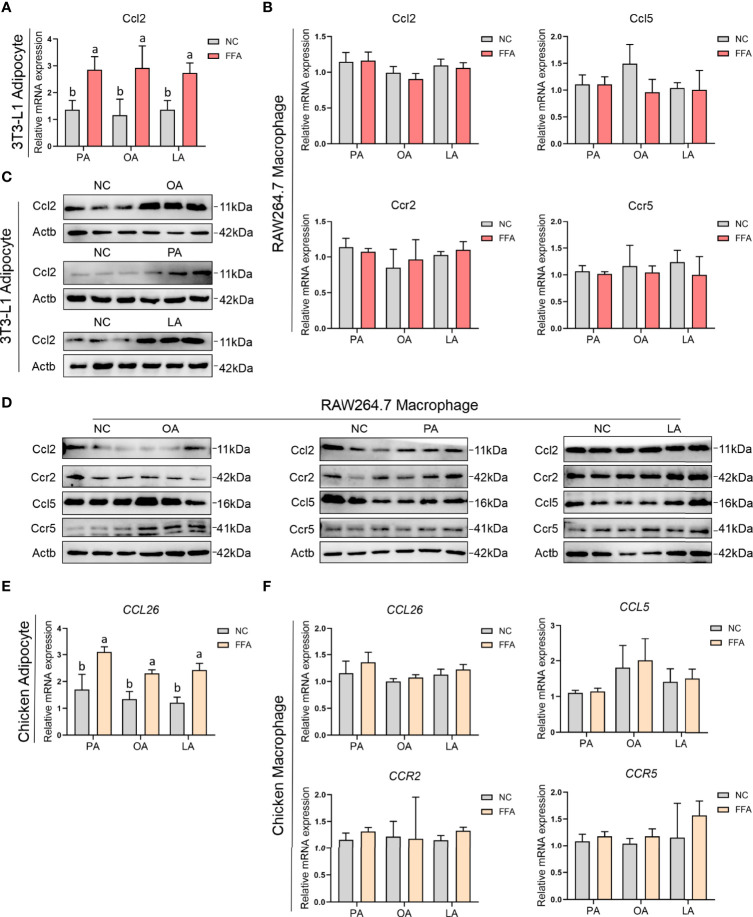
FFA-induced adipocyte and macrophage chemokines and their receptors expression in chicken and mouse. **(A)** FFA-induced 3T3-L1 adipocyte Ccl2 mRNA expression; **(B)** FFA-induced RAW264.7 macrophage Ccl2, Ccl5, Ccr2, and Ccr5 mRNA expression; **(C)** FFA-induced 3T3-L1 adipocyte Ccl2 protein expression; **(D)** FFA-induced RAW264.7 macrophage Ccl2, Ccl5, Ccr2, and Ccr5 protein expression; **(E)** FFA-induced chicken adipocyte CCL26 mRNA expression; **(F)** FFA-induced chicken macrophage CCL26, CCL5, CCR2, and CCR5 mRNA expression. a,b=*p*<0.05.

To explore the relationship between adipocyte and macrophage chemokines and receptors expression, the mRNA and protein expression levels of *Ccl2* (chicken *CCL26*), *CCL5*, *CCR2*, and *CCR5* were measured by qPCR and Western blotting (**Supplementary Figures S1A–C**). The results in **Figure 11** show that the mRNA and protein expression levels of *Ccl2* were significantly upregulated after treatment with FFAs condition medium in 3T3-L1 adipocyte; the mRNA expression level of *CCL26* was also significantly upregulated after treatment with FFA condition medium in chicken adipocyte. Besides, there was no difference with FFA condition medium in RAW264.7 and chicken macrophages.

### FFA-Induced Cell Migration and Adipocyte Recruitment of Macrophage

**Figure 12:**
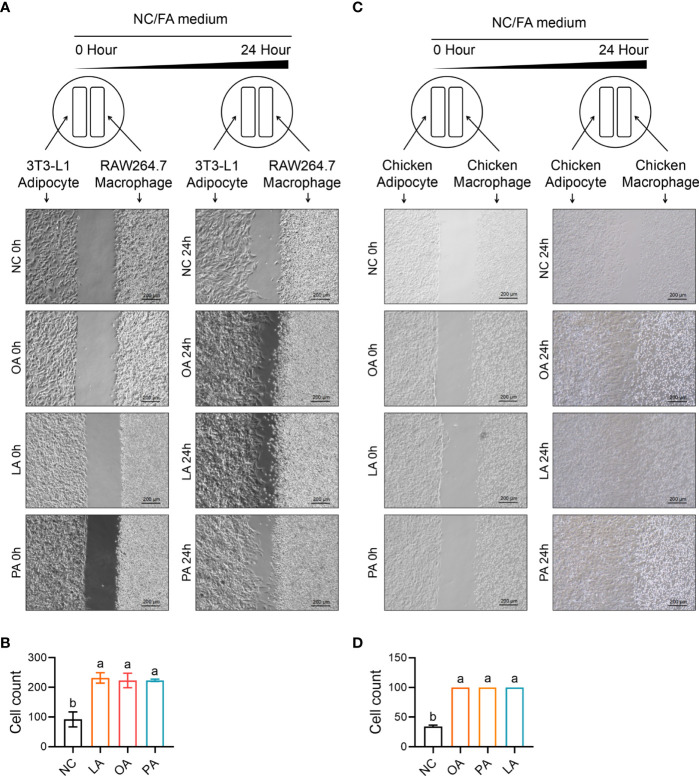
FFA-induced cell migration (insert) between adipocyte and macrophage in chicken and mouse. **(A)** FFA-induced cell migration (insert) between 3T3-L1 adipocyte and RAW264.7 macrophage; **(B)** FFA-induced relative healing area between 3T3-L1 adipocyte and RAW264.7 macrophage for 24 h; **(C)** FFA-induced cell migration (insert) between chicken adipocyte and macrophage; **(D)** FFA-induced relative healing area between chicken adipocyte and macrophage for 24 h a,b=*p*<0.05.

**Figure 13:**
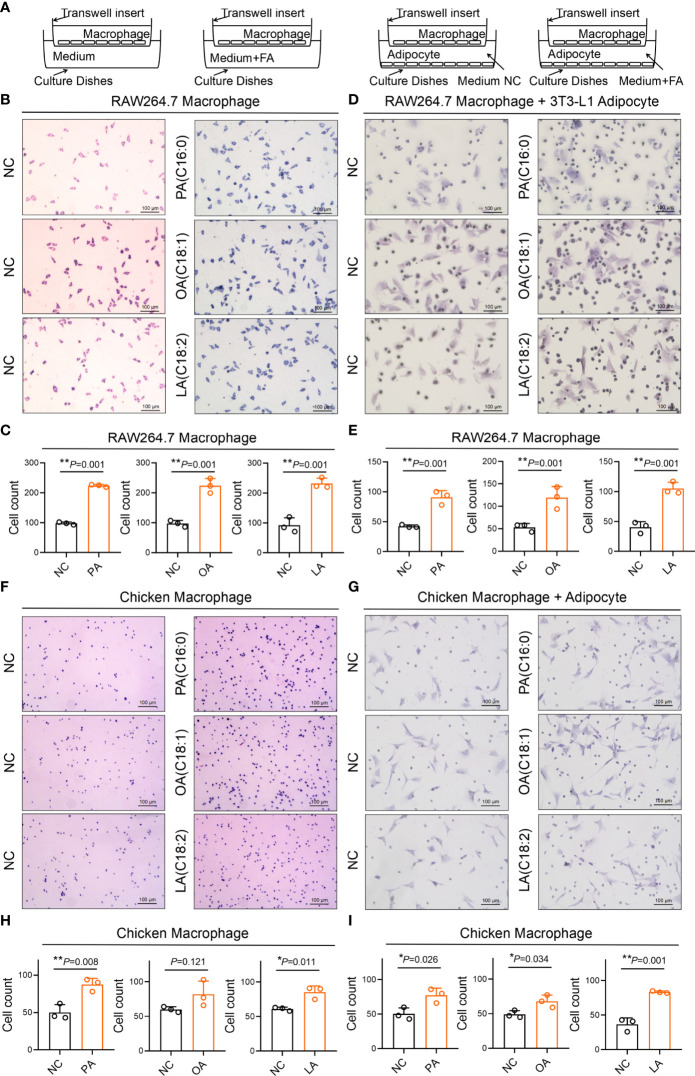
FFA-induced adipocyte recruitment (transwell) of macrophages in chicken and mouse. **(A)** Schematic diagram of FFA-induced adipocyte recruit macrophage in chicken and mouse; **(B, D)** FFA-induced 3T3-L1 adipocyte recruit RAW264.7 macrophage; **(C, E)** FFA-induced 3T3-L1 adipocyte recruit the number of RAW264.7 macrophage in unit area for 24 h; **(F, G)** FFA-induced chicken adipocyte recruit chicken macrophage; **(H, I)** FFA-induced chicken adipocyte recruit the number of chicken macrophage in unit area for 24 h. a,b=*p*<0.05.

Cell migration and recruitment of adipocyte and macrophage were measured by wound healing and transwell assay in the study. As revealed in **Figure 12**, the result shows that cell migration between RAW264.7 macrophage and 3T3-L1 adipocyte was remarkably enhanced by PA, OA, and LA compared with control (*p*<0.05). Meanwhile, the cell migration between chicken macrophage and adipocyte was increased by PA, OA, and LA compared with control (*p*<0.05). The results of the transwell assay showed that FFA significantly promoted the recruitment ability of 3T3-L1 adipocyte compared with control (*p*<0.05). Similarly, FFA significantly promoted the recruitment ability of chicken adipocyte compared with control (*p*<0.05). Besides Ccl2 within 3T3-L1 adipocyte and CCL26 within chicken adipocyte, FFAs themselves can also recruit macrophages (**Figure 13**).

### FFA-Induced Macrophage Foaming

**Figure 14:**
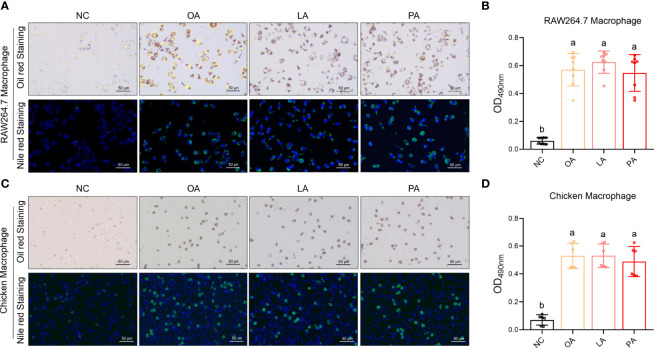
FFA-induced macrophage foaming. **(A)** FFA-induced RAW264.7 macrophage foaming (Oil red and Nile red staining); **(B)** quantization of FFA-induced RAW264.7 macrophage foaming (Oil red staining); **(C)** FFA-induced chicken macrophage foaming (Oil red and Nile red staining); **(D)** quantization of FFA-induced chicken macrophage foaming (Oil red staining). a,b=*p*<0.05.

FFA-induced macrophage foaming was measured by Oil red and Nile red staining. The results showed that three FFAs significantly promoted the foaming level of RAW264.7 macrophage compared with control (*p*<0.05) (**Figures 14A, B** **)**. The results showed that three FFAs significantly promoted the foaming level of chicken macrophage compared with control (*p*<0.05) (**Figures 14C, D** **)**.

## Discussion

In recent years, many researchers have studied the metabolic mechanism of abdominal fat deposition in chickens, ignoring the significance of chick subcutaneous adipose tissue. During the embryonic and post-hatch period, chick subcutaneous adipose tissue appeared earlier than abdominal adipose tissue, and their weight was more than that in abdominal adipose tissue. Our previous study revealed that chicken subcutaneous adipose tissue is another important energy supply tissue during the post-hatch period, but the understanding of chick subcutaneous adipose tissue is still at a low level. Therefore, the purposes of this study were to explore the differences between chick subcutaneous adipose tissue and abdominal adipose tissue during embryonic and post-hatch period through RNA-seq and WGCNA and to explore the cross-talk between adipocytes and macrophages.

The results of RNA-seq of chick subcutaneous adipose tissue in four development stages revealed that the metabolic differences between four development stages were huge. Through qPCR verification, 11 of the 12 DEGs have the same tendency as shown in RNA-seq, which showed that sequencing data has a certain credibility in this study. GO and KEGG enrichment between different development stages showed that the function of chick subcutaneous adipose tissue was related to inflammatory reaction. Combined with the published data on chick abdominal adipose tissue during the embryonic and post-hatch period, we found that there were only 128 common DEGs within E20 vs. D1 between subcutaneous adipose tissue and abdominal adipose tissue. The KEGG enrichment of DEGs (E20 vs. D1) only in abdominal adipose tissue was related to PPARG signal pathway, and the DEGs (E20 vs. D1) only in subcutaneous adipose tissue were related to inflammatory reaction. It revealed that chick subcutaneous adipose tissue seems to be a more active organ than abdominal adipose tissue, and inflammation-related metabolism could have an important relationship with lipid metabolism. Accumulating evidence suggested that subcutaneous and abdominal adipose tissues are differentially associated with metabolic disorders. According to WGCNA of chick subcutaneous and abdominal adipose tissues during the embryonic and post-hatch period, we found that the turquoise module of subcutaneous adipose tissue was enriched in lysosome function and inflammatory reaction, and the brown module of abdominal adipose tissue was related to fatty acid metabolism. It was inferred that the function of chick subcutaneous and abdominal adipose tissues could be largely different in many aspects. The differences between subcutaneous adipose tissue and abdominal adipose tissue in chicks were similar to rodents and human. For example, fatty acid metabolism in subcutaneous adipose tissue is more active, while abdominal adipose tissue has a greater relationship with insulin sensitivity. Interestingly, all bioinformatics analysis of chick subcutaneous adipose tissue points to inflammatory reaction, which was different from the function of subcutaneous adipose tissue in rodents and human. Ten hub genes were identified, and their functions were related to macrophage, which had different mRNA expression patterns from chick abdominal adipose tissue during the embryonic and post-hatch period.

Obesity is characterized by an accumulation of macrophages in adipose, some of which form distinct crown-like structures (CLS) around adipocyte. In addition to macrophages, there are other innate immune cells such as mast cells, neutrophils, eosinophils, group 2 innate lymphoid cells, and adaptive immune cells including T and B cells. To identify the cell type of immune infiltration in chick subcutaneous adipose tissue, IHC (LGALS3), toluidine blue staining, and qPCR were used to confirm the existence of macrophage, mast cells, and T cells, and those results showed that macrophage was the major cell type within chick subcutaneous adipose tissue during the embryonic and post-hatch period. The gene expression programs of adipose tissue macrophages were generally highly divergent and strongly shaped by the tissue environment, with distinct gene modules driving the functional characteristics of locally resident macrophages. It is increasingly being recognized that the nutrient composition present in tissues can influence the phenotypes and inflammatory functions of macrophages. Combined with our previous study, we inferred that the FFA metabolism of subcutaneous adipose tissue was associated with macrophage. As the receptor of FFAs, TLR4 was the first hub gene in chick subcutaneous adipose tissue, once again proving the close relationship between FFA metabolism and macrophage function.

Significantly, chickens do not have *CCL2* gene, and we have investigated the structure of the genomic organization of *CCL2* and *CCL5* gene cluster in chicken and representative species. Based on the phylogenetic tree of *CCL* gene family in adipose tissue of human, mouse, and chicken, *CCL26* in chicken was more closely related to *CCL2* than *CCL26* in mammals. The expression pattern of *Ccl2* (chicken *CCL26*), *CCL5*, *CCR2*, and *CCR5* showed that *CCL26* could be the major chemokine in chicken adipocyte as the status of *CCL2* in mammal adipocyte.

As the three major fatty acids in egg and chick subcutaneous adipose tissue, PA, OA, and LA were used to evaluate the effect of FFA-induced chemotactic migration between adipocyte and macrophage. Three FFA-induced chemotactic migration in mouse and chick adipocytes demonstrated that FFAs increased the secretion of *Ccl2* (chicken *CCL26*) and enhanced the ability of macrophages recruitment. However, FFA-induced chemotactic migration in mouse and chick macrophage showed that FFAs did not affect the secretion of *Ccl2* (chicken *CCL26*) and *CCL5* in macrophage, demonstrating that FFA-mediated macrophage recruitment only existed in adipocytes. This phenotype may be related to protecting chick subcutaneous adipose tissue from excessive immune infiltration or related to the process of macrophages leaving adipose tissue.

There were various interaction patterns between adipocytes and macrophages in adipose tissue and were precisely regulated by the microenvironment of adipose tissue. According to current studies and RNA-seq data in this study, TRIM29 plays an important role in the macrophage activation of adipose tissue, especially in viral infection and inflammation (37, 38). The mRNA expression of TRIM29 in E14 was significantly higher than that in E20, D1, and D9 in chick subcutaneous adipose tissue (**Supplementary Table S3**), which indicated that TRIM29 was the controller of macrophage in adipose tissue; the release of this signal allows macrophages to rely on their high plasticity to perform their specific functions in adipose tissue, including cleaning up damaged adipocytes and protecting adipose tissue from inflammation reaction.

Accumulation of extracellular lipids, as occurring upon excessive cell death or lipidolysis, is a common feature of partial inflammation lipid metabolism disorders. Some studies showed that macrophages had the ability to transport cholesterol, which have strongly correlated with atherosclerosis. It is speculated that the function of macrophages in chick subcutaneous adipose tissue could be related to FFAs transport. Our finding demonstrated that FFAs could be stored in macrophages in large quantities for a short time, which had a similar phenotype with cholesterol-induced macrophage foaming in atherosclerosis. Direct diffusion is the main transport mode of short-chain fatty acids across the membrane, while long-chain fatty acids are mainly dependent on the transport of carrier proteins. In this mode, excessive fatty acid accumulation between cells could be a potential inflammatory risk. Lipidolysis could be one of the driving factors in the cross-talk between adipocyte and macrophage (39). The ability of macrophages to absorb fatty acids combined with their unique cell migration capacity may be an important medium to help lipid transport in adipose tissues (40). It could be confirmed by the negative correlation between CCL2–CCR2 axis and body weight (41). Substantial evidence is still lacking for these speculations, and further studies are needed to prove them. Furthermore, FFA-mediated cross-talk between adipocyte and macrophage also has certain limitations in this study. The gene expression programs of adipose tissue macrophages polarization are generally highly divergent and strongly shaped by the tissue environment, including adipocytokine, cell debris, apoptosis body, and hormone.

## Conclusion

**Figure 15:**
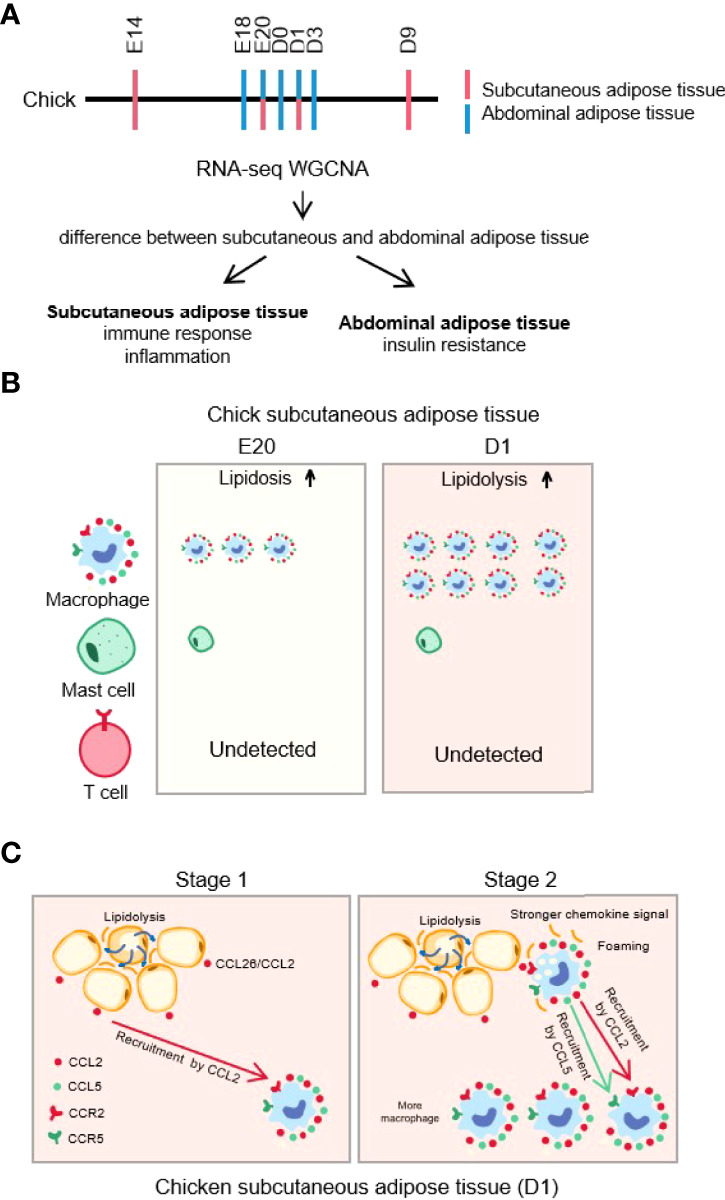
Graphical abstract. **(A)** Transcriptome sequencing and analysis protocol of chick subcutaneous and abdominal adipose during the embryonic and post-hatch period; **(B)** immune infiltration of chick subcutaneous adipose tissue between E20 and D1; **(C)** FFA-induced *Ccl2* (Chicken *CCL26*) secretion is crucial in determining fat depot-selective adipose tissue macrophage (ATM) infiltration.

In this study, combined with our previous study, we investigated the differences between chick subcutaneous adipose tissue and abdominal adipose tissue during the embryonic and post-hatch period by enrichment analysis and WGCNA and found that macrophage was associated with the process of lipid homeostasis. We demonstrated that FFA-induced Ccl2 (chicken CCL26) secretion is crucial in determining fat depot-selective adipose tissue macrophage (ATM) infiltration (**Figure 15**). These findings may have multiple important implications for understanding macrophage biology with chick subcutaneous adipose tissue during the embryonic and post-hatch period.

## Data Availability Statement

The datasets presented in this study can be found in online repositories. The names of the repository/repositories and accession number(s) can be found below: https://www.ncbi.nlm.nih.gov/, PRJNA811769.

## Ethics Statement

The animal study was reviewed and approved by Institutional Animal Care and Use Committee of Northwest A&F University.

## Author Contributions

HZ, MW, and XT analyzed the data; HZ wrote the manuscript; HZ, XT, MW, QL, XY, and WZ collected the samples; QL performed the qPCR; XY and WZ reviewed and edited the manuscript; XS designed the experiment. All authors contributed to the interpretation of the results and writing of the manuscript. All authors contributed to the article and approved the submitted version.

## Funding

Project supported by National Natural Science Foundation of China (31972557).

## Conflict of Interest

The authors declare that the research was conducted in the absence of any commercial or financial relationships that could be construed as a potential conflict of interest.

## Publisher’s Note

All claims expressed in this article are solely those of the authors and do not necessarily represent those of their affiliated organizations, or those of the publisher, the editors and the reviewers. Any product that may be evaluated in this article, or claim that may be made by its manufacturer, is not guaranteed or endorsed by the publisher.

## Supplementary Material

Supplementary Table S1 List of the primers used in this study

Supplementary Table S2 Quality control summary of RNA-seq data

Supplementary Table S3 DEGs list

Supplementary Table S4 Subcutaneous fat different stages KEGG enrichment

Supplementary Table S5 Abdominal fat different stages KEGG enrichment

Supplementary Table S6 Subcutaneous fat VS Abdominal fat E20-D1 genes list.

Supplementary Table S7 Subcutaneous fat VS Abdominal fat E20-D1 KEGG and GO enrichment.

Supplementary Table S8 modules gene lists in abdominal fat and subcutaneous fat by WGCNA.

Supplementary Table S9 GO and KEGG enrichment of modules in different stage subcutaneous fat by WGCNA.

Supplementary Table 10 GO and KEGG enrichment of modules in different stage abdominal fat by WGCNA.

Supplementary Figure S1 The expression of chemokines and their receptors in adipocyte and macrophage. **(A)**: Relative mRNA expression of Ccl2, Ccl5, Ccr2 and Ccr5 in RAW264.7 macrophage and 3T3-L1 adipocyte; **(B)**: Relative protein expression of Ccl2, Ccl5, Ccr2 and Ccr5 in RAW264.7 macrophage and 3T3-L1 adipocyte; **(C)**: Relative mRNA expression of CCL26, CCL5, CCR2 and CCR5 in chicken macrophage and adipocyte. **P*<0.05, ***P*<0.01.

## Abbreviations

ATM, adipose tissue macrophage; WAT, white adipose tissue; BAT, brown adipose tissue; UCP1, uncoupling protein 1; PPARγ, peroxisome proliferator-activated receptor gamma; CPT1a, carnitine palmitoyltransferase 1A; WGCNA, weighted gene coexpression network analysis; E, embryonic; D, day; DEGs, differentially expressed genes; HE, hematoxylin and eosin; IHC, immunohistochemistry; PA, palmitic acid; OA, oleic acid; LA, linoleic acid; qPCR, quantitative real-time PCR; Galectin 3, LGALS3; SDS, sodium dodecyl sulfate; PBS, phosphate-buffered saline; FBS, fetal bovine serum; CCL, C–C motif chemokine ligand; CCR, C–C motif chemokine receptor; HRP, horseradish peroxidase; MAD, median absolute deviation; SEM, standard error of the mean; PCA, principal component analysis; GO, Gene Ontology; KEGG, Kyoto Encyclopedia of Genes and Genomes; CLS, crown-like structures.
