# Supplementary material for: RNA-seq Based Transcriptome Analysis Reveals The Cross-Talk of Macrophage and Adipocyte of Chicken Subcutaneous Adipose Tissue during The Embryonic and Post-Hatch Period
Source: Front Immunol. 2022 Jul 15;13:889439. doi: 10.3389/fimmu.2022.889439 (PMC9334849; doi:10.3389/fimmu.2022.889439)
Supplement: Supplementary Table S1 — List of the primers used in this study [file Table_1.docx]

**Table S1** List of the primers used in this study

| **Genes** | **Accession number** | **Sequencing (5'-3')** | **Efficiency** | **Species** | **Test item** |
| --- | --- | --- | --- | --- | --- |
| gga-*TLR4* | [NM_001030693.1](https://www.ncbi.nlm.nih.gov/nuccore/NM_001030693.1) | F: tggatctttcaaggtgccaca | 1.901 | Chicken | Hub-gene verification |
|  |  | R: agtgtccgatgggtaggtca |  |  |  |
| gga-*MAPK1* | [NM_204150.1](https://www.ncbi.nlm.nih.gov/nuccore/NM_204150.1) | F: ccaacctctcctacatcggc | 1.830 | Chicken | Hub-gene verification |
|  |  | R: tcatgcctgaagcgcagtaa |  |  |  |
| gga-*TLR7* | [NM_001011688.2](https://www.ncbi.nlm.nih.gov/nuccore/NM_001011688.2) | F: tttacatggcccaccaacga | 1.860 | Chicken | Hub-gene verification |
|  |  | R: gcatttttcagacgctgcca |  |  |  |
| gga-*CD80* | [NM_001079739.1](https://www.ncbi.nlm.nih.gov/nuccore/NM_001079739.1) | F: cccaccccacagctgtaaat | 1.957 | Chicken | Hub-gene verification |
|  |  | R: ctgctctcaccccctttcag |  |  |  |
| gga-*STAT1* | [NM_001012914.1](https://www.ncbi.nlm.nih.gov/nuccore/NM_001012914.1) | F: tggtaccagctacagcaactt | 1.844 | Chicken | Hub-gene verification |
|  |  | R: atacgttgttggctgcgtgt |  |  |  |
| gga-*TLR1A* | [NM_001007488.4](https://www.ncbi.nlm.nih.gov/nuccore/NM_001007488.4) | F: agctgccgacatcctttgaa | 1.881 | Chicken | Hub-gene verification |
|  |  | R: aataagtacaggaatttaggaacgc |  |  |  |
| gga-*CD40* | [NM_204665.2](https://www.ncbi.nlm.nih.gov/nuccore/NM_204665.2) | F: tggtgaaggtgaaagggacg | 1.790 | Chicken | Hub-gene verification |
|  |  | R: gtgtgcaccaggcagtagat |  |  |  |
| gga-*CD86* | [NM_001037839.1](https://www.ncbi.nlm.nih.gov/nuccore/NM_001037839.1) | F: tccagcggaggttatccaga | 1.877 | Chicken | Hub-gene verification |
|  |  | R: tgctgctctccaaggtgaag |  |  |  |
| gga-*MAPK12* | [XM_001233061.6](https://www.ncbi.nlm.nih.gov/nuccore/XM_001233061.6) | F: ccaggcatacggacagtgag | 1.854 | Chicken | Hub-gene verification |
|  |  | R: agaggcctccccgttatcat |  |  |  |
| gga-*CMPK2* | [XM_015284945.3](https://www.ncbi.nlm.nih.gov/nuccore/XM_015284945.3) | F: ttatgctgcgggcaactaca | 1.899 | Chicken | RNA seq data verification |
|  |  | R: taagcagctgtgctatgcca |  |  |  |
| gga-*CXCL14* | [NM_204712.2](https://www.ncbi.nlm.nih.gov/nuccore/NM_204712.2) | F: cttggctgacgtaggactgg | 1.894 | Chicken | RNA seq data verification |
|  |  | R: tgcaggcaggtgtgaaatct |  |  |  |
| gga-*DGAT2* | XM_040661934.1 | F: gttggattgccagaggtgga | 1.802 | Chicken | RNA seq data verification |
|  |  | R: ccaaagcagaaaagctcccg |  |  |  |
| gga-*INPP5D* | [XM_025153529.2](https://www.ncbi.nlm.nih.gov/nuccore/XM_025153529.2) | F: tgaactaccgtgtggagcag | 1.808 | Chicken | RNA seq data verification |
|  |  | R: tcaaaacggtaggtgggagc |  |  |  |
| gga-*LCP2* | NM_204701.1 | F: gcttgctgaaggaggtcaca | 1.851 | Chicken | RNA seq data verification |
|  |  | R: tcacctccgaacgataggga |  |  |  |
| gga-*MARCO* | NM_204736.2 | F: ctgcccacttgtatgctcca | 1.936 | Chicken | RNA seq data verification |
|  |  | R: cacacgtctgagctccagtt |  |  |  |
| gga-*MX1* | [NM_204609.1](https://www.ncbi.nlm.nih.gov/nuccore/NM_204609.1) | F: gtgcctctgcgatcaggct | 1.883 | Chicken | RNA seq data verification |
|  |  | R: catgctgctgcctcatcct |  |  |  |
| gga-*RANBP3L* | XM_015277518.3 | F: cattgcgaagctgtgctgag | 1.885 | Chicken | RNA seq data verification |
|  |  | R: gatccactgctgctcacttg |  |  |  |
| gga-*REM1* | XM_015296468.3 | F: gggagctctggcaacatcat | 1.835 | Chicken | RNA seq data verification |
|  |  | R: agggtttccccattgtaccg |  |  |  |
| gga-*RNASE6* | [NM_205259.2](https://www.ncbi.nlm.nih.gov/nuccore/NM_205259.2) | F: tgcatctggatggcaccttt | 1.862 | Chicken | RNA seq data verification |
|  |  | R: tcaaagcgttagggggaacc |  |  |  |
| gga-*TSPO2* | XM_418037.6 | F: agatcccggggtggaataca | 1.855 | Chicken | RNA seq data verification |
|  |  | R: ccgaagcataaagcgtgtcg |  |  |  |
| gga-*ZC3HAV1* | NM_001012938.1 | F: ggagctccacaagagggaaa | 1.814 | Chicken | RNA seq data verification |
|  |  | R: gcgatctgcatgagaaggct |  |  |  |
| gga-*CCL26* | XM_415780.6 | F: ttcagatggcctacccacaac | 1.878 | Chicken | Chemokine |
|  |  | R: gactcctcggggtttacaca |  |  |  |
| gga-*CCL5* | NM_175827.2 | F: atgactgccgtagctgtgtc | 1.840 | Chicken | Chemokine |
|  |  | R: agcagcacacggttgtatca |  |  |  |
| gga-*CCR2* | NM_001045835.1 | F: atgttgcaactcacctgtgc | 1.844 | Chicken | Chemokine receptor |
|  |  | R: tgtcgtccacttcttcagtcaa |  |  |  |
| gga-*CCR5* | NM_001271141.1 | F: gaagacgacgacacagtctga | 1.859 | Chicken | Chemokine receptor |
|  |  | R: ccatggaaatgggcaggtct |  |  |  |
| gga-*IL6R* | NM_001044675.2 | F: cgcctgctggtggaaga | 1.808 | Chicken | Macrophage marker gene |
|  |  | R: tccattgtgaacctgcgctt |  |  |  |
| mmu-*CCL2* | NM_011333.3 | F: gtgctgaccccaagaaggaa | 1.824 | Mouse | Chemokine expression |
|  |  | R: gtgctgaagaccttagggca |  |  |  |
| mmu-*CCL5* | NM_013653.3 | F: catatggctcggacacca | 1.851 | Mouse | Chemokine expression |
|  |  | R: acacacttggcggttcct |  |  |  |
| mmu-*CCR2* | NM_009915.2 | F: tttgcaactgcctctttcct | 1.897 | Mouse | Chemokine receptor |
|  |  | R: cttctgtccctgcttcatcc |  |  |  |
| mmu-*CCR5* | NM_009917.5 | F: atggattttcaagggtcagttcc | 1.876 | Mouse | Chemokine receptor |
|  |  | R: ctgagccgcaatttgtttcac |  |  |  |
| mmu-*ACTB* | NM_007393.5 | F: cacgatggaggggccggactcatc | 1.888 | Mouse | Reference genes |
|  |  | R: taaagacctctatgccaacacagt |  |  |  |
| gga-*CTSG* | [XM_423728.6](https://www.ncbi.nlm.nih.gov/nuccore/XM_423728.6) | F: ACACTGCTTGGAGGGAAACAT | 1.839 | Chicken | Mast cell identification |
|  |  | R: GAGCTTGACCTTCGCTGTCA |  |  |  |
| gga-*LGALS3* | [NM_001302800.2](https://www.ncbi.nlm.nih.gov/nuccore/NM_001302800.2) | F: CCCCGTTTCAAGGAAGACCA | 1.923 | Chicken | Macrophage identification |
|  |  | R: CAGAGCACCTGGAGCTTGAA |  |  |  |
| gga-*CD4* | [NM_204649.2](https://www.ncbi.nlm.nih.gov/nuccore/NM_204649.2) | F: GAGGGAGGAAAAGAGGTTTGC | 1.808 | Chicken | T cell identification |
|  |  | R: TTGCTGAGCCATAATGGGGG |  |  |  |
| gga-*CD8A* | [NM_205235.1](https://www.ncbi.nlm.nih.gov/nuccore/NM_205235.1) | F: GCACAGGACCAGGGGACATA | 1.871 | Chicken | T cell identification |
|  |  | R: TCTCACTGCTTGTTCCTGGC |  |  |  |
| gga-*ACTB* | NM_205518.1 | F: GTGTGATGGTTGGTATGGGC | 1.944 | Chicken | Reference genes |
|  |  | R: CTCTGTTGGCTTTGGGGTTC |  |  |  |
