# Supplementary material for: RNA-seq Based Transcriptome Analysis Reveals The Cross-Talk of Macrophage and Adipocyte of Chicken Subcutaneous Adipose Tissue during The Embryonic and Post-Hatch Period
Source: Front Immunol. 2022 Jul 15;13:889439. doi: 10.3389/fimmu.2022.889439 (PMC9334849; doi:10.3389/fimmu.2022.889439)
Supplement: Supplementary Table S2 — Quality control summary of RNA-seq data [file Table_2.docx]

**Table S2** Quality control summary of RNA-seq data

| **Terms** | **Total Reads**  **Count(#)** | **Q20 Bases**  **Ratio(%)** | **Q30 Bases**  **Ratio(%)** | **GC Bases**  **Ratio(%)** | **Total mapped** | **Uniquely mapped** |
| --- | --- | --- | --- | --- | --- | --- |
| E14_1 | 40341004 | 97.21% | 89.66% | 49.14% | 94.42% | 92.53% |
| E14_2 | 50089866 | 97.57% | 90.60% | 48.99% | 94.49% | 92.80% |
| E14_3 | 46715352 | 97.61% | 90.75% | 49.43% | 94.40% | 92.36% |
| E20_1 | 56198292 | 97.55% | 90.50% | 48.67% | 95.28% | 93.83% |
| E20_2 | 54661800 | 97.62% | 90.70% | 48.52% | 95.26% | 93.85% |
| E20_3 | 50910034 | 97.53% | 90.47% | 48.56% | 95.26% | 93.84% |
| D1_1 | 54357640 | 97.38% | 90.02% | 49.21% | 94.44% | 93.05% |
| D1_2 | 41584052 | 97.58% | 90.57% | 48.69% | 94.71% | 93.37% |
| D1_3 | 48793994 | 96.61% | 87.79% | 48.86% | 94.40% | 93.06% |
| D9_1 | 52088104 | 97.41% | 90.10% | 48.46% | 94.75% | 93.47% |
| D9_2 | 54271476 | 97.66% | 90.84% | 48.33% | 94.76% | 93.45% |
| D9_3 | 47544720 | 97.28% | 89.67% | 48.29% | 94.92% | 93.65% |
