# Supplementary figures and images for: RNA-seq Based Transcriptome Analysis Reveals The Cross-Talk of Macrophage and Adipocyte of Chicken Subcutaneous Adipose Tissue during The Embryonic and Post-Hatch Period
Source: Front Immunol. 2022 Jul 15;13:889439. doi: 10.3389/fimmu.2022.889439 (PMC9334849; doi:10.3389/fimmu.2022.889439)

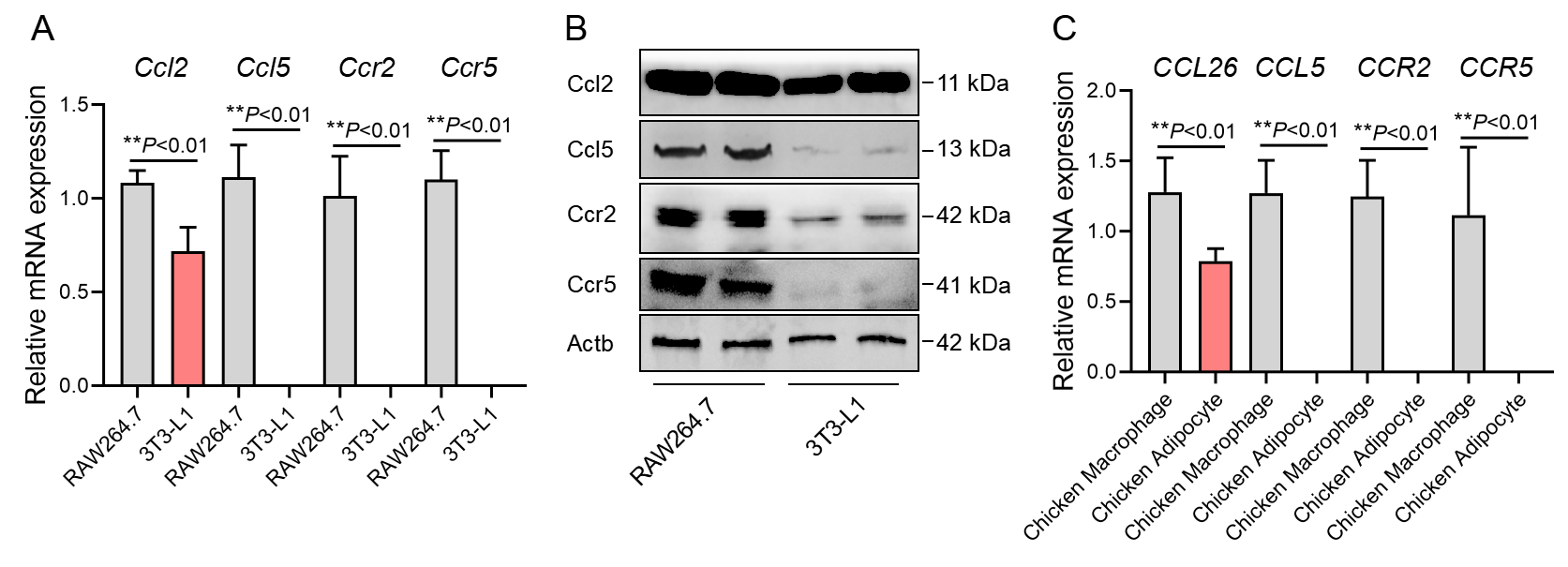

Supplement: Supplementary Figure S1 — The expression of chemokines and their receptors in adipocyte and macrophage. (A): Relative mRNA expression of Ccl2, Ccl5, Ccr2 and Ccr5 in RAW264.7 macrophage and 3T3-L1 adipocyte; (B): Relative protein expression of Ccl2, Ccl5, Ccr2 and Ccr5 in RAW264.7 macrophage and 3T3-L1 adipocyte; (C): Relative mRNA expression of CCL26, CCL5, CCR2 and CCR5 in chicken macrophage and adipocyte. *P<0.05, **P<0.01. [file Image_1.png]
